# Prognostic factors for multi-organ dysfunction in pediatric oncology patients admitted to the pediatric intensive care unit

**DOI:** 10.3389/fonc.2023.1192806

**Published:** 2023-07-12

**Authors:** Marijn Soeteman, Marta F. Fiocco, Joppe Nijman, Casper W. Bollen, Maartje M. Marcelis, Ellen Kilsdonk, Edward E. S. Nieuwenhuis, Teus H. Kappen, Wim J. E. Tissing, Roelie M. Wösten-van Asperen

**Affiliations:** ^1^ Princess Máxima Center for Pediatric Oncology, Utrecht, Netherlands; ^2^ Mathematical Institute, Leiden University, Leiden, Netherlands; ^3^ Department of Pediatric Intensive Care, Wilhelmina Children’s Hospital/University Medical Center Utrecht, Utrecht, Netherlands; ^4^ Department of Pediatrics, Wilhelmina Children’s Hospital/University Medical Center Utrecht, Utrecht, Netherlands; ^5^ Department of Anesthesiology, Wilhelmina Children’s Hospital/University Medical Center Utrecht, Utrecht, Netherlands; ^6^ Department of Pediatric Oncology, University of Groningen, University Medical Center Groningen, Groningen, Netherlands

**Keywords:** pediatric oncology, intensive care unit, multi-organ dysfunction, critical care, prognosis

## Abstract

**Background:**

Pediatric oncology patients who require admission to the pediatric intensive care unit (PICU) have worse outcomes compared to their non-cancer peers. Although multi-organ dysfunction (MOD) plays a pivotal role in PICU mortality and morbidity, risk factors for MOD have not yet been identified. We aimed to identify risk factors at PICU admission for new or progressive MOD (NPMOD) during the first week of PICU stay.

**Methods:**

This retrospective cohort study included all pediatric oncology patients aged 0 to 18 years admitted to the PICU between June 2018 and June 2021. We used the recently published PODIUM criteria for defining multi-organ dysfunction and estimated the association between covariates at PICU baseline and the outcome NPMOD using a multivariable logistic regression model, with PICU admission as unit of study. To study the predictive performance, the model was internally validated by using bootstrap.

**Results:**

A total of 761 PICU admissions of 571 patients were included. NPMOD was present in 154 PICU admissions (20%). Patients with NPMOD had a high mortality compared to patients without NPMOD, 14% and 1.0% respectively. Hemato-oncological diagnosis, number of failing organs and unplanned admission were independent risk factors for NPMOD. The prognostic model had an overall good discrimination and calibration.

**Conclusion:**

The risk factors at PICU admission for NPMOD may help to identify patients who may benefit from closer monitoring and early interventions. When applying the PODIUM criteria, we found some opportunities for fine-tuning these criteria for pediatric oncology patients, that need to be validated in future studies.

## Introduction

The simultaneous dysfunction of multiple organ systems plays a pivotal role in the mortality of children admitted to the pediatric intensive care unit (PICU) (1). Multiple organ dysfunction (MOD) is defined as two or more concurrent organ dysfunctions (1–3). While the term multiple organ dysfunction syndrome (MODS) has traditionally been used, it was recently posited that this term should be selectively applied to patients with a shared underlying mechanism that affects multiple organ systems simultaneously (4). MOD can be categorized in new MOD, defined as MOD in patients who have single or no organ dysfunction on PICU admission, and progressive MOD, defined as additional dysfunctional organ systems in patients who already meet MOD criteria at admission (5).

In children, the risk factors for developing MOD include sepsis, major trauma, severe hypoxemia, and young age (e.g., infancy) (6, 7). The number of dysfunctional organ systems is associated with mortality, with each additional failing organ system increasing the risk of death (7–10). Pediatric oncology patients are particular at high risk for MOD due to the aggressive cancer pathophysiology and intensive treatment regimens, that may lead to organ infiltration, systemic toxicity, and immunosuppression (11). Similarly to general pediatric patients, MOD plays a significant role in the high morbidity and mortality of these patients (12). Early recognition and intervention in organ dysfunction may provide the potential to modify its course and prevent further deterioration (13–16). In adult oncology patients, it was shown that early interventions in deteriorating patients improved both short- and long-term outcomes (14, 15). Therefore, identifying risk factors for MOD at start of the PICU admission could provide opportunities for intensified monitoring and early interventions, which may ultimately reduce morbidity and mortality in critically ill pediatric oncology patients (12, 16, 17). Despite the important role of MOD in PICU morbidity and mortality, risk factors for MOD in pediatric oncology patients have not yet been identified.

In this study, we aimed to identify risk factors at PICU admission for MOD during the first week of PICU stay in pediatric oncology patients. Recently, the Pediatric Organ Dysfunction Information Update Mandate (PODIUM) evidence-based pediatric organ dysfunction criteria were published (12); this is the first study in pediatric oncology patients using these criteria. In addition, fine-tuning of these criteria for pediatric oncology patients may be needed, as they frequently experience organ dysfunction as a result of their oncological treatment. This dysfunction may not necessarily indicate MOD. Therefore, the second objective of this study was to assess whether adjusting the PODIUM criteria for pediatric oncology patients would reveal different risk factors for MOD.

## Methods

We performed a retrospective cohort study between June 1, 2018 and June 1, 2021, at an 18-bed PICU of the Wilhelmina Children’s Hospital, that is shared with the adjacent Princess Máxima Center, an 80-bed national referral center for pediatric oncology. All pediatric oncology patients with International Classification of Diseases in Oncology (ICD-O) diagnosis of pediatric malignancy (morphology code 1, 2 or 3) aged 0 to 18 years admitted to the PICU were eligible for inclusion. Patients without consent for the use of clinical data were excluded. The study was approved by the ethical review board of our hospital (IRB protocol number 16-572/C).

### Assessment of organ dysfunction

We classified organ dysfunction based on the PODIUM criteria (18) (**Table 1** and **Supplementary Table 1**). Clinical data were extracted from the electronic health records and comprised patient characteristics, organ dysfunction in the 24 hours preceding PICU admission, and clinical time series with a frequency of 1 measurement per minute (vital signs and mechanical ventilator data), laboratory results, observations (e.g. Glasgow Coma scores), vasoactive medication, and fluid balance data. Additional data for organ dysfunction, e.g., cardiopulmonary resuscitation, encephalopathy and gastro-intestinal perforation, were acquired from free text fields in clinical or imaging reports through text-mining. In applying the PODIUM criteria, we made assumptions based on clinical expertise to get from a high frequency dataset to the classification of (concurrent) organ dysfunction, including handling measurement errors and missing data. Detailed information on the assessment of the PODIUM criteria is provided in the **Supplementary Material**. Single organ dysfunction was classified based on the PODIUM criteria within 1-hour windows, and the number of concurrent organ dysfunctions was classified within each 24-hour window.

**Table 1:** Assessment of the PODIUM and PONC-PODIUM criteria.

Organ system*	PODIUM criteria	PONC-PODIUM criteria adjustments
Neurologic	Glasgow Coma Scale (GCS) ≤ 8 Cornell Assessment of Pediatric Delirium (CAPD) score ≥ 9	
Respiratory	In patients on respiratory support but not invasively ventilated, i.e. on either high flow nasal cannula (HFNC), non-rebreathing mask (NRM) or non-invasive ventilation): o PaO_2_/FiO_2_ ratio ≤ 300 o SpO_2_/FiO_2_ ratio ≤ 264 o Non-invasive ventilation for ventilatory failure In invasively ventilated patients: o Oxygenation index (OI) ≥ 4 to ≤ 16 o OI >= 16 o Oxygen saturation index (OSI) ≥ 5 to < 12.3 o OSI ≥ 12.3	Only **severe** respiratory dysfunction; - Invasive ventilation with OI ≥ 16 and/or OSI ≥ 12.3
Cardiovascular	Cardiac arrest HR > 2 SD above normal for age o 0–7 d: HR > 180 beats/min o > 1 wk to 1 m: HR > 180 beats/min o > 1 m to < 1 y: HR > 180 beats/min o 6 y to < 13 y: HR > 150 beats/min o 13 y to < 18 y: HR > 130 beats/min SBP > 2 SD above normal for age o 0–7 d: SBP <50 mm Hg o > 1 wk to 1 m: SBP < 70 mm Hg o > 1 m to < 1 y: SBP < 75 mm Hg o 1 y to < 6 y: SBP < 75 mm Hg o 6 y to < 13 y: SBP < 80 mm Hg o 13 y to < 18 y: SBP < 80 mm Hg Vasoactive-inotropic score ≥ 5 Serum lactate ≥ 3 mmol/L Echo cardiographic estimation of left ventricular ejection fraction (LVEF) < 50%	Only **severe** cardiovascular dysfunction in case it was graded; - Resuscitation; or - At least 2 out of 5 of the following criteria present at the same time: HR > 2 SD above normal for age; SBP > 2 SD above normal for age, vasoactive-inotropic score ≥ 5, serum lactate ≥ 5 mmol/L, echo cardiographic estimation of LVEF < 30%;
Renal criteria	- Urine output < 0.5 mL/kg/h for ≥ 6 hours and < 12 hours with concomitant serum creatinine increase 1.5 – 1.9 times baseline or ≥ 26.5 µmol/L increase. - Urine output < 0.5 mL/kg/h for ≥ 12 hours - Serum creatinine increase ≥ 2 times baseline - eGFR < 35 mL/min/1.73 m2 (and not age < 30 days) - Fluid overload ≥ 20% – starting 48 hours after start PICU admission - Initiation of continuous renal replacement therapy (CRRT)	- Oliguria for < 0.5 mL/kg/h for ≥ 6 hours **or** concomitant serum creatinine increase 1.5 – 1.9 times baseline **or** ≥ 26.5 µmol/L increase; or - Serum creatinine increase ≥ 2 times baseline; or - Fluid overload of **10%** from PICU admission onwards; or - eGFR < 35 mL/min/1.73; or - Initiation of renal replacement therapy
Gastrointestinal	Bowel perforation or pneumatosis intestinalis on plain abdominal film, CT or MRI	
Hepatic	o Biochemical evidence of acute liver injury (defined as aspartate aminotransferase > 100 IU/L, alanine aminotransferase > 100 IU/L, gamma-glutamyl transferase > 100 IU/L, total bilirubin > 85.5 µmol/L, or direct bilirubin > 34.2 µmol/L) with prothrombin time (PT) ≥ 15 secs or international normalize ratio (INR) ≥ 1.5 and hepatic encephalopathy o Biochemical evidence of acute liver injury with PT ≥ 20 secs or INR ≥ 2.0	
Hematology	Platelet count < 30 10E9/L or 50% decrease from baseline Hemoglobin < 4.3 mmol/L Leucocytes < 3.0 10E9/L	Only **new dysfunction** throughout PICU stay was included, defined as: - Platelet count < 30 10E9/L (30 000 cells/µL) or 50% decrease from baseline; **or** - Hemoglobin < 4.3 mmol/L
Coagulation	In the absence of liver dysfunction, a combination of ≥ 2 of the following criteria: o Platelet count < 30 10E9/L o INR > 1.5 o Fibrinogen 1.5 g/L o D-dimer > 5 µg/mL (= upper limit of normal)	Platelet count **< 30** 10E9/L (<30 000 cells/µL), and other coagulation criteria were classified according to the original PODIUM criteria.
Endocrine	Blood glucose ≥ 8.3 mmol/L or < 2.8 mmol/L	
Immunology	Peripheral absolute neutrophil count < 0.5 10E9/L	Only **new dysfunction** throughout PICU stay was included, defined as: - Peripheral absolute neutrophil count < 0.5 10E9/L (< 500 cells/µL) or if missing: leucocyte count < 1.0 10E9/L (< 1000 cells/µL)

The main adjustments compared to the original PODIUM criteria are depicted in bold.

*In case an organ system is not displayed, it is classified according to the original PODIUM criteria, see **Supplementary Table S2**.

PONC-PODIUM, pediatric oncology Pediatric Organ Dysfunction Information Update Mandate; NPMOD, new or progressive organ dysfunction; OI, oxygenation index; OSI, oxygenation saturation index; HR, heart rate; SBP, systolic blood pressure; SD, standard deviation; LVEF, left ventricular ejection fraction; eGFR, estimated glomerular filtration rate.

We assessed presence of organ dysfunction at PICU admission (baseline) by evaluating all relevant laboratory values and free text data in the 24 hours prior to and the first three hours of PICU admission. Missing data were classified as no organ dysfunction at PICU baseline. For further details on assessment of the organ dysfunction criteria, see **Supplementary Table 2**.

### Adjustments in PODIUM criteria for pediatric oncology patients

Although some specific criteria for oncology patients are included in the PODIUM criteria, we proposed additional considerations for these patients since some laboratory variables may reflect side-effects of the cancer treatment instead of organ dysfunction in the context of MOD. We therefore adjusted some criteria for this specific patient population: the pediatric oncology (PONC) PODIUM criteria (**Table 1**).

Invasive ventilation and the use of vasoactive medication are associated with increased PICU mortality in pediatric oncology patients (19). Therefore, we used the thresholds of severe respiratory dysfunction, i.e., invasive ventilation and an oxygenation index of ≥ 16 or an oxygenation saturation index of ≥ 12.3. For cardiovascular dysfunction, we used the severe threshold for lactate and left ventricular ejection fraction (LVEF).

Considering the renal criteria, it was shown that patients with a fluid overload greater than 10% were 6 times more likely to die during PICU admission than those with less than or equal to 10% fluid overload (20). Moreover, oliguria is often not present in pediatric oncology patients with acute kidney injury (AKI) (20). We therefore adjusted the criteria for renal dysfunction: oliguria was not required and a fluid overload > 10%, instead of 20%, was used directly from the start of PICU admission onwards (as opposed to starting 48 hours after admission).

Since hematological and immunological dysfunction at baseline are less relevant due to the idiopathic nature of these in oncology patients and likely does not represent dysfunction due to critical illness, we excluded the leukocyte criterion from hematological dysfunction and only included hematological or immunological dysfunction that was newly developed during PICU stay for the classification of NPMOD. In classifying coagulation dysfunction, we used the platelet count threshold for pediatric oncology patients (i.e., < 30 10E9/L or < 30 000 cells/µL).

### Primary outcome: new or progressive multi-organ dysfunction

The primary outcome was new or progressive MOD (NPMOD). New MOD was defined as no MOD at baseline and the concurrent dysfunction of at least 2 organs. Progressive MOD was defined as MOD (i.e., concurrent dysfunction of at least 2 organ systems) at baseline, and the development of one or more additional concurrent organ dysfunction(s).

### Statistical analysis

A multivariable logistic regression model was used to estimate the association between covariates and the outcome. Covariates at baseline of PICU admission were selected based on literature and expert opinion. The included covariates encompassed diagnosis category (i.e., hemato-oncological, solid tumor or neuro-oncological); hematopoietic stem cell transplantation; neutropenia at baseline; a composite covariate of sepsis and/or infection (bacterial or fungal (21)); high-flow oxygen therapy preceding PICU admission; the number of organ dysfunctions at baseline (categorized into 0, 1 or ≥ 2), unplanned PICU admission, and previous relevant PICU admission (i.e., a previous PICU admission that was either unplanned or had a protracted course). See **Supplementary Material** for a detailed description of the covariates.

We analyzed the first week of PICU admission, or up to discharge within seven days, whichever event first occurred. We assessed the outcome NPMOD based on both the original and our PONC-PODIUM criteria, to determine whether adjustments of the organ dysfunction criteria for pediatric oncology population yielded different significant risk factors. In addition, we performed a subgroup analysis of only unplanned PICU admissions to identify possible different significant risk factors for NPMOD. A multivariable logistic regression model was used to estimate the association between covariates and the outcome, including the same covariates as before except for unplanned PICU admission. The outcome NPMOD within one week based on both original PODIUM criteria and PONC-PODIUM criteria was assessed.

To study the predictive performance of the model, internal validation was performed by using Efron’s bootstrap (i.e. resampling the dataset 500 times) (22). Statistical analyses were performed using R-statistical software (23), version 4.2.1 (2022–06–23)., see **Supplementary Material** for associated packages.

## Results

A total of 761 PICU admissions of 571 patients were included. **Table 2** reports the clinical characteristics of the PICU admissions. The median age [interquartile range] at PICU admission was 6.0 [2.7 – 12.8] years. The cohort included 25% hemato-oncological patients, 35% solid tumor patients, 40% neuro-oncology patients, and 2% had a hematopoietic stem cell transplantation (HSCT) in the year preceding PICU admission. Among the 761 PICU admissions, 288 (38%) were unplanned admissions. Neuro-oncology and solid tumor patients most often had planned postoperative PICU admissions (89% and 67% respectively), whereas hemato-oncology patients largely required unplanned PICU admissions (93%). Data of at least 2 organ systems were available at baseline in 744 of 761 PICU admissions (98%) for the classification of MOD at baseline.

**Table 2:** Clinical and demographic characteristics of PICU admissions overall and by occurrence of NPMOD (defined according to PODIUM criteria).

Characteristic	Total PICU admissions (n = 761)	PICU admissions without NPMOD (n = 607)	PICU admissions with NPMOD (n = 154)
*General characteristics per PICU admission*			
Age at admission (years), median [IQR]	6.0 [2.7 – 12.8]	6.5 [3.0 – 13.1]	4.0 [1.5 – 11.0]
Female sex, n (%)	351 (46)	265 (44)	86 (56)
PICU admission reason, n (%)			
Planned post-operative care	473 (62.2)	444 (73.1)	29 (18.8)
Respiratory failure	106 (13.9)	49 (8.1)	57 (37.0)
Sepsis	40 (5.3)	25 (4.1)	15 (9.7)
Neurological deterioration	36 (4.7)	27 (4.4)	9 (5.8)
Cardiovascular failure	33 (4.3)	20 (3.3)	13 (8.4)
Renal failure	7 (0.9)	1 (0.2)	6 (3.9)
Liver failure	2 (0.3)	1 (0.2)	1 (0.6)
Unplanned post-operative care	24 (3.2)	16 (2.6)	8 (5.2)
Other	40 (5.3)	24 (4.0)	16 (10.4)
*Covariates*			
Oncological diagnosis groups			
Hemato-oncological	190 (25.0)	101 (16.6)	89 (57.8)
Solid tumor	268 (35.2)	225 (37.1)	43 (27.9)
Brain/CNS tumor	303 (39.8)	281 (46.3)	22 (14.3)
HSCT, n (%)	16 (2.1)	5 (0.8)	11 (7.1)
Infection or sepsis at baseline, n (%)	100 (13.1)	52 (8.6)	48 (31.2)
Neutropenia at baseline, n (%)	82 (10.8)	47 (7.7)	35 (22.7)
HFNC preceding admission, n (%)	86 (11.3)	46 (7.6)	40 (26.0)
Previous relevant PICU admission, n (%)	104 (13.7)	67 (11.0)	37 (24.0)
Unplanned PICU admission, n (%)	288 (37.8)	163 (26.9)	125 (81.2)
Number of failing organs at baseline, n (%)			
0	471 (61.9)	416 (68.5)	49 (31.8)
1	159 (20.9)	117 (19.3)	45 (29.2)
>= 2	131 (17.2)	74 (12.2)	60 (39.0)
***Outcome***			
Maximum number of concomitantly failing organs during first week of PICU stay			
0	346 (45.5)	346 (57.3)	0 (0)
1	209 (27.5)	209 (34.6)	0 (0)
2	78 (10.2)	28 (4.6)	50 (32.5)
3	56 (7.4)	16 (2.6)	40 (26.0)
4	34 (4.5)	5 (0.8)	29 (18.8)
>= 5	38 (4.9)	3 (0.5)	35 (22.3)
PICU length of stay (days), median [IQR]	0.9 [0.8 – 2.5]	0.9 [0.7 – 1.4]	5.0 [2.1 – 10.0]
PICU mortality, n (%)	28 (3.7)	6 (1.0)	22 (14.3)

IQR, interquartile range; CNS, central nervous system; HSCT, hematopoietic stem cell transplantation; HFNC, high flow nasal cannula oxygen therapy; NPMOD, new or progressive multi-organ dysfunction; PICU, paediatric intensive care unit.

### NPMOD classified according to original PODIUM criteria

**Figure 1:**
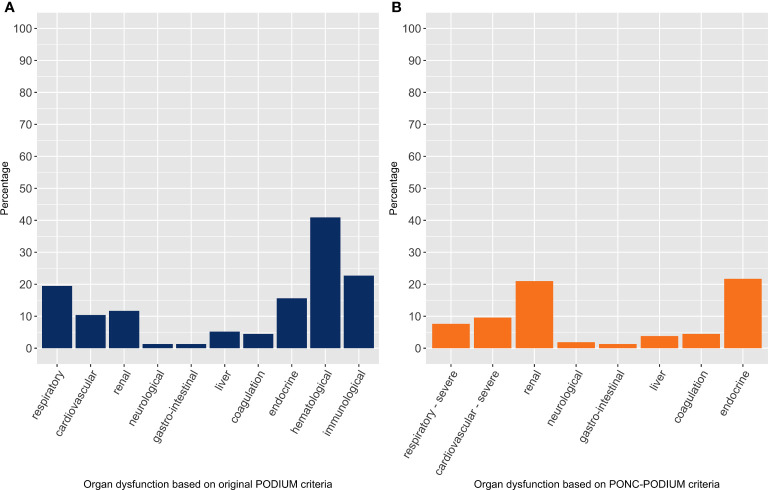
Organ dysfunction at PICU baseline in all PICU admissions with new or progressive multi-organ dysfunction, per organ system with percentage of failing organ system. The left panel **(A)** considers organ dysfunction based on the original PODIUM criteria, whereas the right panel **(B)** considers organ dysfunction based on the PONC-PODIUM criteria, thus adjusted for pediatric oncology patients.

NPMOD was present in 154 PICU admissions (20%). The PICU mortality was 4% in all PICU admissions, 1% in the group without NPMOD, and 14% in the group with NPMOD. In the PICU admissions where patients developed NPMOD, the three most frequently failing organ systems at PICU baseline included hematological (41%), immunological (23%) and respiratory (20%) dysfunction (see **Figure 1A**).

The results of the univariate and multivariable model are displayed in **Table 3**. Hemato-oncological diagnosis, number of failing organs at baseline and unplanned PICU admissions were significantly associated with NPMOD in the multivariable model. Internal validation of the model yielded a c-index of 0.81, indicating a reasonable discriminative ability. The calibration plot showed an overall good calibration, with an index-corrected slope of 0.93.

**Table 3:** Results of the univariate and multivariable logistic regression model, with estimated odds ratio (OR) along with the 95% confidence interval (CI), for outcome of new or progressive multi organ dysfunction (NPMOD) - defined according to the PODIUM criteria.

Covariate	Univariate OR (95% CI)	Multivariable OR (95% CI)
Oncological diagnosis groups		
Hemato-oncological	11.19 [6.71 – 18.67]	**2.23 [1.14 – 4.36]**
Solid tumor	2.33 [1.36 – 3.98]	1.29 [0.70 – 2.37]
Brain/CNS tumor	*Reference*	*reference*
HSCT, n (%)	9.26 [3.17 – 27.07]	1.66 [0.52 – 5.22]
Infection or sepsis at baseline, n (%)	4.83 [3.10 – 7.53]	1.63 [0.93 – 2.88]
Neutropenia at baseline	3.50 [2.16 – 5.66]	0.46 [0.21 – 1.02]
HFNC preceding admission	4.27 [2.67 – 6.84]	1.17 [0.67 – 2.03]
Previous relevant PICU admission	2.54 [1.63 – 3.99]	1.07 [0.63 – 1.83]
Unplanned PICU admission	11.74 [7.55 – 18.27]	**5.82 [3.37 – 10.07]**
Number of failing organs at baseline		
0	*Reference*	*reference*
1	3.26 [2.07 – 5.14]	**2.18 [1.30 – 3.67]**
>= 2	6.88 [4.38 – 10.81]	**2.39 [1.18 – 4.83]**

CNS, central nervous system; HSCT, hematopoietic stem cell transplantation; HFNC, high flow nasal cannula oxygen therapy.

Significant covariates in the model are in bold.

### NPMOD classified according to PONC-PODIUM criteria

Using the PONC-PODIUM criteria, NPMOD was present in 157 PICU admissions (21%), see **Supplementary Table S3**. Applying these adjusted criteria revealed a different top three of frequently failing organ systems at PICU baseline, namely endocrine (22%), renal (21%), and severe cardiovascular dysfunction (10%) (**Figure 1B**). In the multivariable model, we found the same significant risk factors for NPMOD including hemato-oncological diagnosis, number of failing organs at baseline and unplanned PICU admission (**Supplementary Table S4**).

### Unplanned PICU admissions

**Figure 2:**
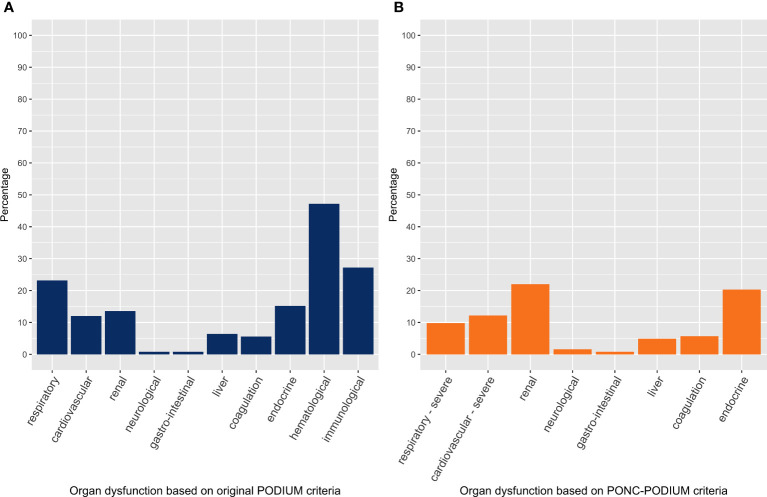
Organ dysfunction at PICU baseline in unplanned PICU admissions with new or progressive multi-organ dysfunction. The left panel **(A)** considers organ dysfunction based on the original PODIUM criteria, whereas the right panel **(B)** considers organ dysfunction based on the PONC-PODIUM criteria.

We performed a subgroup analysis including only the unplanned admissions (**Table 4**). NPMOD according to the original PODIUM criteria was present in 125 unplanned PICU admissions (43%). Respiratory failure, sepsis and neurological deterioration were the three major PICU admission reasons for unplanned PICU admission. PICU mortality rate was slightly higher compared to the total cohort, 4% in the patients without NPMOD and 17% in patients with NPMOD. The most frequently failing organ systems at admissions were similar to what was found in the total cohort, including hematological dysfunction (47%), immunological dysfunction (27%), and respiratory dysfunction (23%) (**Figure 2A**). In the multivariable logistic regression model, the number of failing organs at PICU baseline was significantly associated with NPMOD (**Table 5**).

**Table 4:** Clinical and demographic characteristics of unplanned PICU admissions, by occurrence of new or progressive multi organ dysfunction (defined according to PODIUM criteria).

Characteristic	Unplanned PICU admissions (n = 288)	Unplanned PICU admissions without NPMOD (n = 163)	Unplanned PICU admissions with NPMOD (n = 125)
***General characteristics per PICU admission***			
Age at admission (years), median [IQR]	5.8 [2.3 – 13.1]	7.2 [2.6– 13.5]	4.1 [1.9 – 11.4]
Female sex, n (%)	143 (49.7)	70 (42.9)	73 (58.4)
PICU admission reason, n (%)			
Respiratory failure	106 (36.8)	49 (30.1)	57 (45.6)
Sepsis	40 (13.9)	25 (15.3)	15 (12.0)
Neurological deterioration	36 (12.5)	27 (16.6)	9 (7.2)
Cardiovascular failure	33 (11.5)	20 (12.2)	13 (10.4)
Renal failure	7 (2.4)	1 (0.6)	6 (4.8)
Liver failure	2 (0.7)	1 (0.6)	1 (0.8)
Unplanned post-operative care	24 (8.3)	16 (9.8)	8 (6.4)
Other	40 (13.9)	24 (14.7)	16 (12.8)
***Covariates***			
Oncological diagnosis groups			
Hemato-oncological	168 (58.3)	84 (51.5)	84 (67.2)
Solid tumor	88 (30.6)	56 (34.4)	32 (25.6)
Brain/CNS tumor	32 (11.1)	23 (14.1)	9 (7.2)
HSCT, n (%)	16 (5.6)	5 (3.1)	11 (8.8)
Infection or sepsis at baseline, n (%)	86 (29.9)	40 (24.5)	46 (36.8)
Neutropenia at baseline, n (%)	75 (26.0)	41 (25.2)	34 (27.2)
HFNC preceding admission, n (%)	79 (27.4)	40 (24.5)	39 (31.2)
Previous relevant PICU admission, n (%)	71 (24.7)	38 (23.3)	33 (26.4)
Number of failing organs at baseline, n (%)			
0	107 (37.2)	75 (46.0)	32 (25.6)
1	65 (22.6)	30 (18.4)	35 (28.0)
>= 2	116 (40.3)	58 (35.6)	58 (46.4)
***Outcome***			
Maximum number of concomitantly failing organs during first week of PICU stay			
0	59 (45.5)	59 (36.2)	0 (0)
1	58 (27.5)	58 (35.6)	0 (0)
2	53 (10.2)	23 (14.1)	30 (24.0)
3	48 (7.4)	15 (9.2)	33 (26.4)
4	33 (4.5)	5 (3.1)	28 (22.4)
>= 5	37 (12.8)	3 (1.8)	34 (27.2)
PICU length of stay (days), median [IQR]	2.2 [1.0 – 6.0]	1.4 [0.7 – 2.8]	5.6 [2.2 – 10.9]
PICU mortality, n (%)	27 (9.4)	6 (3.7)	21 (16.8)

IQR, interquartile range; CNS, central nervous system; HSCT, hematopoietic stem cell transplantation; HFNC, high flow nasal cannula oxygen therapy; NPMOD, new or progressive multi-organ dysfunction; PICU, paediatric intensive care unit.

**Table 5:** Results of the univariate and multivariable logistic regression model, with estimated odds ratio (OR) along with the 95% confidence interval (CI), for outcome of new or progressive multi organ dysfunction in unplanned PICU admissions (defined according to the PODIUM criteria).

Covariate	Univariate OR (95% CI)	Multivariable OR (95% CI)
Oncological diagnosis groups		
Hemato-oncological	2.56 [1.12 – 5.85]	1.89 [0.78 – 4.58]
Solid tumor	1.46 [0.60 – 3.54]	1.24 [0.49 – 3.12]
Brain/CNS tumor	*Reference*	*reference*
HSCT, n (%)	3.05 [1.03 – 9.01]	1.76 [0.55 – 5.57]
Infection or sepsis at baseline, n (%)	1.79 [1.08 – 2.98]	1.66 [0.90 – 3.03]
Neutropenia at baseline	1.11 [0.65 – 1.89]	0.45 [0.20 – 1.02]
HFNC preceding admission	1.39 [0.83 – 2.34]	1.21 [0.69 – 2.14]
Previous relevant PICU admission	1.18 [0.69 – 2.02]	0.97 [0.54 – 1.74]
Number of failing organs at baseline		
0	*Reference*	*reference*
1	2.73 [1.44 – 5.18]	**2.19 [1.13 – 4.28]**
>= 2	2.34 [1.35 – 4.07]	**2.55 [1.17 – 5.66]**

CNS, central nervous system; HSCT, hematopoietic stem cell transplantation; HFNC, high flow nasal cannula oxygen therapy.

Significant covariates in the model are in bold.

Using our PONC-PODIUM criteria in the cohort of unplanned admissions, NPMOD was present in 123 unplanned PICU admissions (43%) (**Supplementary Table S5**). In the unplanned admissions with NPMOD, the most frequent failing organ systems at admission included renal dysfunction (22%), endocrine dysfunction (20%), and severe cardiovascular dysfunction (12%) (**Figure 2B**). Consistent with the application of the original PODIUM criteria, the multivariable model showed that the number of failing organs was a significant risk factor associated with the occurrence of NPMOD (**Supplementary Table S6**).

## Discussion

This is the first study using the recently published PODIUM criteria for organ dysfunction (18) in pediatric oncology patients to identify risk factors for new or progressive multi-organ failure during the first week of PICU admission. Considering all PICU admissions, we found that hemato-oncological diagnosis, unplanned PICU admission and number of failing organs at PICU baseline were independent risk factors. In the subgroup of the unplanned PICU admissions, we found that the number of failing organs at PICU baseline was independently associated with NPMOD.

Our finding that hemato-oncological diagnosis is a significant risk factor for developing NPMOD is in line with other studies showing that hemato-oncological patients have greater illness severity at PICU admission, experience multi-organ failure more often, require more PICU resources and have a higher PICU mortality compared to solid tumor patients (11, 12, 24, 25). The high risk for organ dysfunction may be attributed to the combination of generally more dose-intense chemotherapy and glucocorticoids, that may result in increased toxic side-effects and profound and prolonged myelosuppression (11, 12, 26). Yet, upon analysis in only unplanned PICU admissions, we found that although a hemato-oncological diagnosis was associated with NPMOD in the univariate analysis, it was not a significant risk factor for NPMOD in the multivariable analysis.

Surprisingly, neutropenia was not a significant risk factor both in the total cohort and cohort of unplanned admissions. Some other studies in adult and pediatric oncology patients also failed to demonstrate an association of neutropenia with worse outcomes, in a multivariable analysis (27–29). Advances in the diagnosis and treatment of infections, the prescription of prophylactic antibiotics and antifungals, and antibiotic stewardship may have limited the role of neutropenia in worse outcome in critically ill oncology patients. A recent study including only pediatric hemato-oncology patients with unplanned PICU admissions showed that neutropenia was an independent risk factor for PICU mortality (30). Our study differs in that we also included patients with a solid or a brain or central nervous system tumor. The degree of multi-organ dysfunction during PICU admission is a significant prognostic factor for PICU mortality in pediatric oncology patients (12). We found that the presence of MOD already at PICU admission is an independent risk factor for progressive MOD, in both the total cohort as in the subgroup including only unplanned PICU admissions. These findings are in line with a study in general pediatric patients, showing that the presence of MOD on day 1 of PICU admission was associated with death or poor neurologic outcome (8). Our finding that PICU mortality in patients with NPMOD in the unplanned admissions was only slightly higher compared to the total cohort including also planned post-operative patients, emphasizes the pivotal role of MOD in the outcome of these patients. Early recognition of deteriorating organ functions before PICU admission followed by early initiation of appropriate treatment may be important to reduce morbidity and mortality in critically ill pediatric oncology patients (12, 16, 31, 32).

In the present study, we tailored the PODIUM criteria to pediatric oncology patients. The adjustments in renal criteria can be valuable to prevent missing AKI, as it was shown that AKI, even stage 1, is significantly associated with short- and long-term complications in critically ill children (33). Second, according to PODIUM, neutropenia is a classifier for dysfunction of two different organ systems (hematologic and immunologic), where we included dysfunction that is more likely to be part of a shared underlying pathway for MOD (e.g. in sepsis) instead of chemotherapeutic treatment. Furthermore, we found a high percentage of endocrine dysfunction. The threshold for glucose ≥ 8.3 mmol/L (150 mg/dL) might be a threshold at which particularly hemato-oncology patients are easily flagged, due to steroid-induced adrenal insufficiency or hyperglycemia (34). This threshold could be considered to be fine-tuned and validated in future studies.

Using our PONC-PODIUM criteria, we found different organ systems that frequently failed at PICU admissions. Endocrine, renal and severe cardiovascular dysfunction emerged as the most frequently failing organ systems in patients who develop NPMOD. This finding may merely have implications for early surveillance at the inpatient ward, prior to PICU admission. Particularly renal and cardiovascular dysfunction can be recognized in an early phase, and timely, appropriate interventions may potentially halt progression to irreversible organ damage. For example, the development of acute kidney injury (AKI) can be monitored at the ward, and substitution or adjustments of nephrotoxic medication and prevention of fluid overload can be easily implemented (35). This may lead to decreased AKI rates and better outcomes (33, 35). In addition, closely monitoring the fluid balance and prevention of fluid overload in patients with cardiovascular failure could provide an opportunity to prevent further deterioration.

Our study revealed several challenges in applying predefined criteria for organ dysfunction to a dataset with continuous data at a frequency of 1 minute and interval data. We accounted for measurement errors and missing data. We thereupon defined age-based limits for artefacts in vital signs, carried last observations forward for a limited time defined per variable and classified organ dysfunction within 1-hour timeframes, to minimize that a single value could immediately flag organ dysfunction. Last observation carried forward to deal with missing data was similarly used in a retrospective study on the early prediction of organ dysfunction in children (36). We used the 24 hours preceding PICU admission to classify organ dysfunction at PICU admission. As PODIUM criteria did not incorporate a specific time period required to fulfil the criteria for organ dysfunction, we classified the concurrent number of failing organ systems within 24-hour windows. Yet, for future studies, a validated time period required to fulfil the criteria especially for respiratory and cardiovascular dysfunction may further optimize defining (concurrent) organ dysfunction.

This is the first study including all organ systems of the PODIUM criteria, as we extracted free text field data using an automatized process of text mining with standardized search terms to, for example, identify gastro-intestinal dysfunction. In addition, our study evaluated a PICU cohort that encompasses all subgroups of pediatric oncology patients, including HSCT patients, from a national referral center where oncology care has been nationally centralized.

Our study has several limitations. First, the data retrieved from patients’ medical records were primarily captured for clinical care. Consequently, selective measurements, such as laboratory values only assessed upon clinical suspicion of organ dysfunction, may bias the timing of onset of (multiple) organ dysfunction. Therefore, we summarized to NPMOD within 24-hour-time frames. Second, our study is a single-center study. Consequently, our findings may not be generalizable due to international differences in PICU policies regarding admission and care. Third, we did not have data on morbidity following prior PICU admissions. We therefore defined a relevant prior PICU admission as any prior unplanned admission, or a prior planned admission with a protracted course. For future studies, to assess the effect of a prior PICU admission on the risk of developing NPMOD in a current PICU admission, it would be beneficial to include data on relevant comorbidity following a prior admission. Last, in this retrospective study, we could not differentiate between underlying mechanisms of organ dysfunction and could thus not define MOD syndrome (MODS). The identification of a common underlying pathobiology, such as in MODS, may be helpful to evolve from isolated organ specific to more holistic strategies that target a common pathobiology (4).

This study shows that hemato-oncological diagnosis, number of failing organs and an unplanned admission are significant risk factors at PICU admission for the development of NPMOD in pediatric oncology patients. For future perspectives, we see opportunities to further refine the PODIUM criteria for pediatric oncology patients. Currently, the PODIUM criteria have been validated in general pediatric patients (5), and are yet to be validated in pediatric oncology patients. We provided a first step towards further refinement of these criteria for pediatric oncology patients. Yet, the criteria introduced in this study need to be validated, preferably in a large multi-center cohort incorporating all subgroups of pediatric oncology patients. The results of the present study may help to guide both intensivists and oncologists in risk stratification for critically ill pediatric oncology patients and to identify patients who may benefit from closer monitoring and early interventions at the ward prior to PICU admission.

## Data availability statement

The raw data supporting the conclusions of this article will be made available by the authors, without undue reservation.

## Ethics statement

The studies involving human participants were reviewed and approved by IRB protocol number 16-572/C. Written informed consent to participate in this study was provided by the participants’ legal guardian/next of kin.

## Author contributions

MS, WT and RW-vA designed the project. MS, JN, CB, MM and EK collected the data. MS, MF, and TK conducted the data analyses. MS, MF, JN, EN, TK, WT, and RW-vA drafted the manuscript. MS prepared the tables and figures. MS, MF, TK, WT, and RW-vA had full access to all the data in the study and take responsibility for the integrity of the data and the accuracy of the analysis. All authors contributed to the article and approved the submitted version.
