# Supplementary material for: Prognostic factors for multi-organ dysfunction in pediatric oncology patients admitted to the pediatric intensive care unit
Source: Front Oncol. 2023 Jul 12;13:1192806. doi: 10.3389/fonc.2023.1192806 (PMC10369184; doi:10.3389/fonc.2023.1192806)
Supplement: Supplementary file 1 [file Table_1.docx]

Supplementary Material

Prognostic factors for multi-organ dysfunction in pediatric oncology patients admitted to the pediatric intensive care unit.

Marijn Soeteman, Marta Fiocco, Joppe Nijman, Casper W. Bollen, Maartje M. Marcelis, Ellen Kilsdonk, Edward E.S. Nieuwenhuis, Teus H. Kappen, Wim J.E. Tissing, Roelie M. Wösten-van Asperen*.

*** Correspondence:** Roelie Wösten-van Asperen, r.m.vanasperen@umcutrecht.nl

**Table of contents**

# Supplementary methods – data collection and data cleaning

## Extraction of clinical data…………………………………………………………….…………………………....2

## Assessment and classification of the PODIUM organ dysfunction criteria in the EHR datasets…………………………………………………………………………………………………….…..…….2

**Supplementary Table S1:** Assessment and classification of the PODIUM organ dysfunction criteria in the EHR datasets…………………………………………………………………………………………………………..…...4

## Supplementary Table S2: Detailed description of the covariates, and data extraction and cleaning for the assessment of these covariates in the datasets………………………………………………………...…………..…7

**Supplementary Table S3:** Clinical and demographic characteristics of all PICU admissions, with NPMOD defined by PONC-PODIUM criteria.…...……………………………………………..……………………..….…..8

**Supplementary Table S4** Results of univariate and multivariable logistic regression model, with estimated odds ratio (OR) along with the 95% confidence interval (CI) for the outcome of new or progressive multi organ dysfunction (defined according to the PONC-PODIUM criteria)…......................................................…..…....….9

**Supplementary Table S5:** Clinical and demographic characteristics of only unplanned PICU admissions, where NPMOD has been defined according to PONC-PODIUM criteria………………………………………………...10

**Supplementary Table S6:** Results of univariate and multivariable logistic regression model, with estimated odds ratio (OR) along with the 95% confidence interval (CI) for the outcome of new or progressive multi organ dysfunction in unplanned PICU admissions (defined according to the PONC-PODIUM criteria)…...…..…....….11

**Supplementary Methods**

1. ***Data collection and data cleaning***

*1.1 Extraction of clinical data*

Clinical data pertaining to the period prior to PICU admission were extracted from the electronic health records (EHR; HiX, Chipsoft, Amsterdam, the Netherlands). The extracted datasets include: demographic data - oncological diagnosis, and patient biometrics; previous PICU admissions; bacterial culture results; laboratory values; all free text fields of daily reports of clinicians and radiology reports for defining covariates (e.g., fungal infection) or organ dysfunction (e.g., gastro-intestinal perforation or reduced left ventricular ejection fraction).

Clinical data pertaining to the period of PICU admission were extracted from MetaVision Patient Data Management System (PDMS;iMDsoft,Tel Aviv, Israel). These data include vital signs, ventilator settings, laboratory values, data on procedures (tube, catheter, arterial line), continuous medication, observations (e.g., Glasgow Coma Scale or Cornell Assessment of Pediatric Delirium (CAP-D) score, and fluid balance. An additional dataset on free text field items, such as resuscitation during PICU stay or hepatic encephalopathy was extracted using text mining using standard search terms on the daily clinicians’ reports at the PICU. More details on definition and data extraction of the covariates is provided in Supplementary Table S1.

Patients without consent for the use of their clinical data were excluded from our study. Data cleaning and analyses were performed in R, version 4.2.1., running under MacOS Big Sur, and the following packages were used:  castoRedc 1.0.5,  rms 6.3-0, table1 1.4.2, lubridate 1.9.0, magrittr 2.0.3, tidyverse 1.3.2, ggplot2 3.4.0, sqldf 0.4-11, readxl 1.4.1, tidyr 1.2.1 , stringr 1.4.1, dplyr 1.0.10, xts 0.12.2, zoo 1.8-11, hablar 0.3.0, pacman 0.5.1, quanteda 3.2.4.

- 1. *Assessment and classification of the PODIUM organ dysfunction criteria in the EHR datasets.*

The dataset from the PICU is a clinical time series dataset with a frequency of 1 measurement per minute. It includes vital signs, mechanical ventilator data, laboratory results, observations (e.g. Glasgow Coma scores), inotropic medication, and fluid balance data. In order to populate infrequently measured physiologic data for continuous organ dysfunction labelling, we used carry-forward interpolation, whereby we defined a time period for each variable. As such, ventilator settings were carried forward for 6 hours, lactate and non-invasive systolic blood pressure were carried forward for 1 hour, and all lab values were carried forward for 6 hours, except for blood glucose and creatinine measures that were carried forward for 1 hour and 24 hours, respectively. As some time points were missing in the time series data, for example when the patient was on MRI transport, we accounted for these missing periods by making the dataset a regular 1-minute time series prior to carrying the last observation forward for the predefined amount of hours.

To identify most commonly occurring patterns of artefacts, the vital signs (mainly heart rate and blood pressure) were checked by visual inspection in MetaVision PDMS electronic health care records (by the first author MS and cases of doubt were resolved by a second reviewer TK). We hereby identified thresholds for artefacts in heart rate and blood pressure, erring on the side of caution, and the values defined as artefacts were transformed into missing values. We did not use the p5 or p95 cut-off values as this more likely could eliminate vital signs that are actually real values.

We summarized the time series with a frequency of 1 measurement per minute to 1-hour windows. Lab values and other relevant data from the period preceding PICU admission were joined to the 1-hour dataset, to enable classification of organ dysfunction at PICU baseline. Moreover, additional datasets with the free text field variables for organ dysfunction criteria were joined. We then defined organ dysfunction according to PODIUM criteria in this 1-hour dataset, see Supplementary Table S2.

The 1-hour dataset with organ dysfunction classification per hour was summarized into 24-hour windows, and new or progressive multi-organ dysfunction was classified based on those 24-hour windows. We defined PICU baseline as the period of 24 hours preceding PICU admission, or the first 3 hours of PICU admission – as it may take a short time from start of PICU admission to start supportive therapy for organ dysfunction, for example intubation and mechanical ventilation. After baseline, any additional organ dysfunction was taken into account for defining NPMOD.

**Supplementary Table S1** Assessment and classification of the PODIUM organ dysfunction criteria in the EHR datasets

| **Organ system** | **Criteria that were considered** | **Additional information** |
| --- | --- | --- |
| Neurologic | Glasgow Coma Scale (GCS) <= 8 | GCS where no sedative medications were given, and the patient was not intubated. Post-operative patients without a tube with a GCS <=8 during the first 3 hours of PICU admission were excluded as this was regarded as a post-sedation effect and not a reflection of neurologic dysfunction.  The electroencephalography (EEG) results were not available in a structured format in either the EHR, and therefore were not included as criteria. |
|  | Cornell Assessment of Pediatric Delirium (CAPD) score ≥ 9 |  |
| Respiratory | In patients on respiratory support but not invasively ventilated, i.e. on either high flow nasal cannula (HFNC), non-rebreathing mask (NRM) or non-invasive ventilation):   - PaO_2_/FiO_2_ ratio ≤ 300 - SpO_2_/FiO_2_ ratio ≤ 264 - Non-invasive ventilation for ventilatory failure | As our center does not provide extracorporeal membrane oxygenation (ECMO) for respiratory failure, this was not included as a criterion. However, we have classified these patients with (severe) respiratory failure prior to transfer for ECMO.  For the proposed SpO_2_-based measures, according to PODIUM criteria only oxygen saturation values between 80% and 97% were considered. PaO_2_/FiO_2_, SpO_2_/FiO_2_, OI and OSI were calculated every minute in order to obtain exact ratios, and respiratory dysfunction was classified using the mean value of these ratios / indices per hour. |
|  | In invasively ventilated patients:   - Oxygenation index (OI) ≥ 4 to ≤ 16 - OI >= 16 - Oxygen saturation index (OSI) ≥5 to < 12.3 - OSI ≥ 12.3 |  |
| Cardiovascular | Cardiac arrest* | Data were obtained through automatized text-mining of free text fields of clinicians’ and nurses’ notes of MetaVision PDMS, through the Dutch search terms and regular expressions for “resuscitation”, “CPR”, “thorax compressions”, “heart massage”. |
|  | Heart rate* | First, artefacts were defined per age category, if a 1-minute value was defined as artefact, we made it a missing (NA). We then summarized this to 1-hour windows, if a window had more than 40 out of 60 observations missing, this window was deemed unsuitable for organ dysfunction classification and we made this 1-hour window missing. We then used the mean value per hour for classification of organ dysfunction per hour. |
|  | Systolic blood pressure* | First, artefacts were defined per age category, and artefacts were made missing. Non-invasive systolic blood pressure was interpolated for 1 hour using last observation carried forward. After cleaning of artefacts, invasive and non-invasive blood pressure were combined into one variable, where invasive blood pressure was leading. Organ dysfunction was then classified based on mean value for every 1 hour. |
|  | Vaso-active inotropic score (VIS)* | VIS was calculated every 1 hour, using the maximum value per inotropic medication for that hour. |
|  | Serum lactate >= 3 and < 5 mmol/L*  or serum lactate >= 5 mmol/L* |  |
|  | Echocardiographic estimation of left ventricular ejection fraction (LVEF) < 50%* | Automatized text-mining with the Dutch search terms and regular expressions for “LVH”, “left ventricle”, “ejection” with exclusion of patterns with (“good*\|normal*\|improving*”) was used to extract data on LVEF < 50% or LVEF described as ‘moderate’ or ‘poor’ from pediatric cardiologists’ notes and ultrasound reports. |
|  |  | Central venous oxygenation and serum troponin were not taken into account as these data were not routinely available.  *Cardiovascular dysfunction was classified based on ≥ 2 of the measurements marked with an asterix (*) at the same hour. |
| Renal | Urine output < 0.5 mL/kg/h for ≥ 6 hours and < 12 hours **with** concomitant serum creatinine increase 1.5 – 1.9 times baseline or ≥ 26.5 µmol/L increase | Only urine output in patients with a catheter was taken into account.  Baseline creatinine was the lowest creatinine in 7 days prior to PICU admission, or, if missing, the lowest creatinine in 30 days prior to PICU admission, or, if missing, the lowest creatinine in 90 days prior to PICU admission.  To define renal dysfunction at baseline, the last creatinine values in the 36 hours preceding PICU admission were used (relative to their baseline creatinine values). If a prior serum creatinine was unavailable, the age-and gender-based baseline creatinine levels proposed by the PODIUM renal dysfunction group (1) were used. Height and weight measurements were obtained from HiX EPD in the 60 days before (and for height 30 days after) PICU admission. |
|  | Urine output < 0.5 mL/kg/h for ≥ 12 hours |  |
|  | Serum creatinine increase ≥ 2 times baseline |  |
|  | eGFR < 35 mL/min/1.73 m2 (and not age < 30 days) |  |
|  | Initiation of continuous renal replacement therapy (CRRT) | The start date and time of CRRT was used to define initiation of CRRT. |
|  | Fluid overload ≥ 20% -- starting 48 hours after start PICU admission | Fluid overload was calculated based on input/output, as data on weight during PICU admission was not routinely available. |
| Gastro-intestinal | Bowel perforation or pneumatosis intestinalis on plain abdominal film, CT or MRI | Data were extracted using the Dutch search terms and regular expressions for “gastro-intestinal perforation”, “gut perforation”, “gut ischemia”, “pneumatosis intestinalis” or “free abdominal air” on free text fields of radiology reports of the EHR and free text fields of physicians’ and nurses’ notes during PICU admission from MetaVision PDMS.  Sloughing of gut was not taken into account as data were not available in structured format or text fields of the EHR. |
| Hepatic | - Biochemical evidence of acute liver injury (defined as aspartate aminotransferase > 100 IU/L, alanine aminotransferase > 100 IU/L, gamma-glutamyl transferase > 100 IU/L, total bilirubin > 85.5 µmol/L, or direct bilirubin > 34.2 µmol/L) **with** prothrombin time (PT) > 15 secs or international normalize ratio (INR) > 1.5 **and** hepatic encephalopathy - Biochemical evidence of acute liver injury **with** PT≥20 secs or INR >- 2.0 | Hepatic encephalopathy was extracted using the search terms or regular expressions for “encephalopathy” in HiX EHR to identify patients with hepatic encephalopathy preceding PICU admission and in MetaVision PDMS for hepatic encephalopathy during PICU stay. This rendered 1 PICU admission, in which the patient already had a PT > 20 or INR > 2.0, and was therefore already classified as having hepatic dysfunction.  Lab values in 24 hours preceding PICU admission were used for classification of hepatic dysfunction at PICU baseline. |
| Hematology | Platelet count < 30 10E9/L or 50% decrease from baseline | Baseline thrombocytopenia was defined as lowest value where platelet count < 100 10E9/L in the 24 hours preceding PICU admission.  We used the lowest value in the 24 hours preceding PICU admission for defining hematological dysfunction at PICU baseline. |
|  | Hemoglobin < 4.3 mmol/L |  |
|  | Leucocytes < 3.0 10E9/L |  |
| Coagulation | In the absence of liver dysfunction, a combination of ≥ 2 of the following criteria:   - Platelet count < **30** 10E9/L - INR > 1.5 - Fibrinogen 1.5 g/L - D-dimer > 5 µg/mL (= upper limit of normal) | As we included only pediatric oncology patients, we adjusted for thrombocytopenia by using a platelet count threshold < 30 10E9/L or 50% decrease from thrombocyte baseline, i.e. we used the same criteria as defined in hematological dysfunction by PODIUM. |
| Endocrine | Blood glucose ≥ 8.3 mmol/L or < 2.8 mmol/L | Glucose measurements in the 12 hours preceding PICU admission were used to define endocrine dysfunction at baseline. |
| Immune | Peripheral absolute neutrophil count < 0.5 10E9/L | If neutrophil count was missing, we used leucocyte count < 1.0 10E9/L as a substitute or neutropenia. Lymphocyte count, CD4^+^ T lymphocyte measurements, monocyte HLA-DR expression and ex vivo LPS-induced TNF-α were not taken into account as these data were not routinely available. |

**Supplementary Table S2:** *Detailed description of the covariates, including data extraction and data cleaning*

| **Covariate** | **Definition** |
| --- | --- |
| Oncological diagnosis group | Hemato-oncological, solid tumor or brain / central nervous system tumor. Diagnoses were manually classified into one of the three diagnosis groups. |
| Hematopoietic stem cell transplantation (HSCT) | A HSCT in the year prior to PICU admission. |
| Sepsis or infection | Sepsis: PICU admission reason was classified as sepsis, based on criteria of the 2005 Pediatric Sepsis Consensus Conference (2).  Fungal infection: probable or proven Aspergillus, Mucor mycosis, or invasive Candida infection, according to the European Organization for Research and Treatment of Cancer/Mycoses Study Group (EORTC/MSG) criteria (3).  Bacterial infection: positive bacterial culture with treatment consequences, i.e. no bacterial colonization. All bacterial cultures were cross checked through text mining in the electronic health records. |
| Neutropenia at baseline | Neutropenia (i.e. neutrophil granulocytes < 0.5 10E9/L or if missing leukocytes < 1.0 10E9/L) in the 24 hours preceding PICU admission or the first 3 hours of PICU admission. |
| Previous relevant PICU admission | Per patient, a prior PICU admission that was either unplanned or had a protracted course, i.e. longer than the anticipated PICU stay; for solid tumor resections the anticipated stay was 1 day, for neuro-oncology patients this was manually defined, ranging from 1 to 3 days based on the risk for developing central diabetes insipidus. |
| Unplanned PICU admission | All other PICU admission reasons other than planned post-operative care or a planned admission after a procedure. |
| Number of failing organs at baseline | Organ dysfunction was defined according to PODIUM criteria (4).  The concomitant number of failing organs per 24-hour window was then categorized into:  0 = no failing organs at baseline  1 = 1 failing organ at baseline  2 = 2 or more failing organs at baseline.  Baseline = 24 hours prior to PICU admission and up to first 3 hours of PICU admission. |

**Supplementary Table S3:** Clinical and demographic characteristics of PICU admissions, with NPMOD defined by PONC-PODIUM criteria**.**

| **Characteristic** | **Total PICU admissions**  **(n = 761)** | **PICU admissions without NPMOD**  **(n = 605)** | **PICU admissions with NPMOD**  **(n = 157)** |
| --- | --- | --- | --- |
| ***General characteristics per PICU admission*** | | | |
| Age at admission (years), median [IQR] | 6.0 [2.7 – 12.8] | 6.6 [3.0 – 13.1] | 4.1 [1.5 – 10.8] |
| Female sex, n (%) | 351 (46) | 269 (44.5) | 82 (52.6) |
| PICU admission reason, n (%) |  |  |  |
| Planned post-operative care | 473 (62.2) | 439 (72.7) | 34 (21.7) |
| Respiratory failure | 106 (13.9) | 53 (8.8) | 53 (33.8) |
| Sepsis | 40 (5.3) | 21 (3.5) | 19 (12.1) |
| Neurological deterioration | 36 (4.7) | 28 (4.6) | 8 (5.1) |
| Cardiovascular failure | 33 (4.3) | 18 (3.0) | 16 (10.2) |
| Renal failure | 7 (0.9) | 2 (0.3) | 5 (3.2) |
| Liver failure | 2 (0.3) | 1 (0.2) | 1 (0.6) |
| Unplanned post-operative care | 24 (3.2) | 19 (3.1) | 5 (3.2) |
| Other | 40 (5.3) | 24 (4.0) | 16 (10.2) |
| ***Covariates*** | | | |
| Oncological diagnosis groups |  |  |  |
| Haemato-oncological | 190 (25.0) | 100 (16.6) | 90 (57.3) |
| Solid tumor | 268 (35.2) | 222 (36.8) | 46 (29.3) |
| Brain / CNS tumor | 303 (39.8) | 282 (46.7) | 21 (13.4) |
| HSCT, n (%) | 16 (2.1) | 4 (0.7) | 12 (7.6) |
| Infection or sepsis at baseline, n (%) | 100 (13.1) | 49 (8.1) | 51 (32.5) |
| Neutropenia at baseline, n (%) | 82 (10.8) | 39 (6.5) | 43 (27.4) |
| HFNC preceding admission, n (%) | 86 (11.3) | 43 (7.1) | 43 (27.4) |
| Previous relevant PICU admission, n (%) | 104 (13.7) | 63 (10.4) | 41 (26.1) |
| Unplanned PICU admission, n (%) | 288 (37.8) | 165 (27.3) | 123 (78.8) |
| Number of failing organs at baseline, n (%) |  |  |  |
| 0 | 552 (72.5) | 481 (79.6) | 71 (45.2) |
| 1 | 169 (22.2) | 105 (17.4) | 64 (40.8) |
| ≥ 2 | 40 (5.3) | 18 (3.0) | 22 (14.0) |
| ***Outcome*** |  |  |  |
| Maximum number of concomitantly failing organs during first week of PICU admission |  |  |  |
| 0 | 358 (47.0) | 358 (59.3) | 0 (0) |
| 1 | 234 (30.7) | 234 (38.7) | 0 (0) |
| 2 | 82 (10.8) | 10 (1.7) | 72 (45.9) |
| 3 | 47 (6.2) | 0 (0.0) | 47 (29.9) |
| 4 | 30 (3.9) | 2 (0.3) | 28 (17.8) |
| ≥ 5 | 9 (1.2) | 0 (0) | 10 (6.4) |
| PICU length of stay (days), median [IQR] | 0.9 [0.8 – 2.5] | 0.9 [0.7 – 1.4] | 4.5 [2.0 – 10.3] |
| PICU mortality, n (%) | 28 (3.7) | 7 (1.2) | 21 (13.4) |

*Abbreviations: PONC-PODIUM: pediatric oncology - Pediatric Organ Dysfunction Information Update Mandate; IQR: interquartile range; NPMOD: new or progressive multi-organ dysfunction; OR: odds ratio; 95% CI: 95% confidence interval; CNS: central nervous system; HSCT: hematopoietic stem cell transplantation; HFNC: high flow nasal cannula oxygen therapy; PICU: pediatric intensive care unit.*

**Supplementary Table S4:** Results of univariate and multivariable logistic regression model, with estimated odds ratio (OR) along with the 95% confidence interval (CI) for the outcome of new or progressive multi organ dysfunction (defined according to the PONC-PODIUM criteria).

| **Covariate** | **Univariate OR (95% CI)** | **Multivariable OR (95% CI)** |
| --- | --- | --- |
| Oncological diagnosis groups |  |  |
| Hemato-oncological | 12.09 [7.13 – 20.47] | **2.64 [1.33 – 5.26]** |
| Solid tumor | 2.78 [1.61 – 4.80] | 1.63 [0.89 – 2.98] |
| Brain / CNS tumor | *reference* | *reference* |
| HSCT, n (%) | 12.41 [3.95 – 39.05] | 2.65 [0.76 – 9.23] |
| Infection or sepsis at baseline, n (%) | 5.45 [3.50 – 8.49] | 1.46 [0.82 – 2.60] |
| Neutropenia at baseline | 5.46 [3.39 – 8.81] | 1.09 [0.58 – 2.04] |
| HFNC preceding admission | 4.92 [3.08 – 7.86] | 1.40 [0.79 – 2.49] |
| Previous relevant PICU admission | 3.03 [1.95 – 4.72] | 1.42 [0.83 – 2.44] |
| Unplanned PICU admission | 9.62 [6.32 – 14.64] | **3.92 [2.27 – 6.76]** |
| Number of failing organs at baseline |  |  |
| 0 | *reference* | *reference* |
| 1 | 4.13 [2.77 – 6.15] | **3.61 [2.27 – 5.73]** |
| >= 2 | 8.28 [4.23 – 16.20] | **3.00 [1.44 – 6.25]** |

PONC-PODIUM: pediatric oncology - Pediatric Organ Dysfunction Information Update Mandate; NPMOD: new or progressive multi-organ dysfunction; CNS: central nervous system; HSCT: hematopoietic stem cell transplantation; HFNC: high flow nasal cannula oxygen therapy. PICU: pediatric intensive care unit.

**Supplementary Table S5:** Clinical and demographic characteristics of only unplanned PICU admissions, by occurrence of NPMOD (defined according to PONC-PODIUM criteria).

| **Characteristic** | **Unplanned PICU admissions**  **(n = 288)** | **Unplanned PICU admissions without NPMOD**  **(n = 165)** | **Unplanned PICU admissions with NPMOD**  **(n = 123)** |
| --- | --- | --- | --- |
| ***General characteristics per PICU admission*** | | | |
| Age at admission (years), median [IQR] | 5.8 [2.3 – 13.1] | 7.6 [ 2.4– 13.5] | 4.5 [2.1 – 11.3] |
| Female sex, n (%) | 143 (49.7) | 73 (44.2) | 70 (56.9) |
| PICU admission reason, n (%) |  |  |  |
| Respiratory failure | 106 (36.8) | 53 (32.1) | 53 (43.1) |
| Sepsis | 40 (13.9) | 21 (12.7) | 19 (15.4) |
| Neurological deterioration | 36 (12.5) | 28 (17.0) | 8 (6.5) |
| Cardiovascular failure | 33 (11.5) | 17 (10.3) | 16 (13.0) |
| Renal failure | 7 (2.4) | 1 (0.6) | 5 (4.1) |
| Liver failure | 2 (0.7) | 1 (0.6) | 1 (0.8) |
| Unplanned post-operative care | 24 (8.3) | 16 (9.8) | 5 (4.1) |
| Other | 40 (13.9) | 24 (14.5) | 16 (13.0) |
| ***Covariates*** | | | |
| Oncological diagnosis groups |  |  |  |
| Hemato-oncological | 168 (58.3) | 80 (48.5) | 88 (71.5) |
| Solid tumor | 88 (30.6) | 61 (37.0) | 27 (22.0) |
| Brain / CNS tumor | 32 (11.1) | 24 (14.5) | 8 (6.5) |
| HSCT, n (%) | 16 (5.6) | 4 (2.4) | 12 (9.8) |
| Infection or sepsis at baseline, n (%) | 86 (29.9) | 37 (22.4) | 49 (39.8) |
| Neutropenia at baseline, n (%) | 75 (26.0) | 32 (19.4) | 43 (35.0) |
| HFNC preceding admission, n (%) | 79 (27.4) | 37 (22.4) | 42 (34.1) |
| Previous relevant PICU admission, n (%) | 71 (24.7) | 35 (21.2) | 36 (29.3) |
| Number of failing organs at baseline, n (%) |  |  |  |
| 0 | 174 (60.4) | 120 (72.7) | 54 (43.9) |
| 1 | 78 (27.1) | 31 (18.8) | 47 (38.2) |
| >= 2 | 36 (12.5) | 14 (8.5) | 22 (17.9) |
| ***Outcome*** |  |  |  |
| Maximum number of concomitantly failing  organs during first week of PICU stay |  |  |  |
| 0 | 82 (45.5) | 59 (36.2) | 0 (0) |
| 1 | 73 (27.5) | 58 (35.6) | 0 (0) |
| 2 | 53 (10.2) | 23 (14.1) | 30 (24.0) |
| 3 | 42 (7.4) | 15 (9.2) | 33 (26.4) |
| 4 | 29 (4.5) | 5 (3.1) | 28 (22.4) |
| >= 5 | (12.8) | 3 (1.8) | 38 (30.8) |
| PICU length of stay (days), median [IQR] | 2.2 [1.0 – 6.0] | 1.4 [0.7 – 2.8] | 5.8 [2.4 – 12.7] |
| PICU mortality, n (%) | 27 (9.4) | 7 (4.2) | 20 (16.3) |
| IQR: interquartile range; CNS: central nervous system; HSCT: hematopoietic stem cell transplantation; HFNC: high flow nasal cannula oxygen therapy; NPMOD: new or progressive multi-organ dysfunction; PICU: paediatric intensive care unit | | | |

**Supplementary Table S6:** Results of the univariate and multivariable logistic regression model, with estimated odds ratio (OR) along with the 95% confidence interval (CI), for outcome of new or progressive multi organ dysfunction in unplanned PICU admissions (defined according to the PONC-PODIUM criteria).

| **Covariate** | **Univariate OR (95% CI)** | **Multivariable OR (95% CI)** |
| --- | --- | --- |
| Oncological diagnosis groups |  |  |
| Hemato-oncological |  | 1.97 [0.78 - 4.93] |
| Solid tumor |  | 0.96 [0.67 – 2.56] |
| Brain / CNS tumor | *reference* | *Reference* |
| HSCT, n (%) | 4.35 [1.36 – 13.8] | 2.59 [0.75 – 8.98] |
| Infection or sepsis at baseline, n (%) | 2.29 [1.37 – 3.83] | 1.55 [0.84 – 2.87] |
| Neutropenia at baseline | 2.23 [1.31 – 3.81] | 1.19 [0.62 – 2.29] |
| HFNC preceding admission | 1.79 [1.06 – 3.02] | 1.56 [0.86 – 2.83] |
| Previous relevant PICU admission | 1.54 [0.90 – 2.64] | 1.23 [0.67 – 2.25] |
| Number of failing organs at baseline |  |  |
| 0 | *Reference* | *reference* |
| 1 | 3.37 [1.93 – 5.87] | **2.80 [1.56 – 5.03]** |
| >= 2 | 3.49 [1.66 – 7.34] | **2.94 [1.34 – 6.40]** |
| CNS: central nervous system; HSCT: hematopoietic stem cell transplantation; HFNC: high flow nasal cannula oxygen therapy.  Significant covariates in the model are in bold | | |

**References**

1. Fitzgerald JC, Basu RK, Fuhrman DY, Gorga SM, Hassinger AB, Sanchez-Pinto LN, et al. Renal dysfunction criteria in critically ill children: The PODIUM Consensus Conference. *Pediatrics* (2022)149(1 Suppl 1):S66-S73. doi: 10.1542/peds.2021-052888J

2. Goldstein B, Giroir B, Randolph A, International Consensus Conference on Pediatric Sepsis. International pediatric sepsis consensus conference: definitions for sepsis and organ dysfunction in pediatrics. *Pediatr Crit Care* (2005) 6(1):2-8. doi: 10.1097/01.PCC.0000149131.72248.E6

3. Donnelly JP, Chen SC, Kauffman CA, Steinbach WJ, Baddley JW, Verweij PE, et al. Revision and update of the consensus definitions of invasive fungal disease from the European organization for research and treatment of cancer and the mycoses study group education and research consortium. *Clin Infect Dis* (2020) 71(6):1367-76. doi: 10.1093/cid/ciz1008

4. Bembea MM, Agus M, Akcan-Arikan A, Alexander P, Basu R, Bennett TD, et al. Pediatric Organ Dysfunction Information Update Mandate (PODIUM) Contemporary Organ Dysfunction Criteria: Executive Summary. *Pediatrics* (2022) 149(1 Suppl 1):S1-S12. doi: 10.1542/peds.2021-052888B
